# Involvement of co-repressor LUH and the adapter proteins SLK1 and SLK2 in the regulation of abiotic stress response genes in *Arabidopsis*

**DOI:** 10.1186/1471-2229-14-54

**Published:** 2014-02-24

**Authors:** Barsha Shrestha, Bhuwan Guragain, Vaniyambadi V Sridhar

**Affiliations:** 1 Department of Biological Sciences, University of New Orleans, New Orleans, LA 70148, USA

**Keywords:** *Arabidopsis*, Co-repressor, LUH, SLK1, SLK2, Abiotic stress, Histone deacetylase

## Abstract

**Background:**

During abiotic stress many genes that are important for growth and adaptation to stress are expressed at elevated levels. However, the mechanisms that keep the stress responsive genes from expressing under non stress conditions remain elusive. Recent genetic characterization of the co-repressor LEUNIG_HOMOLOG (LUH) and transcriptional adaptor proteins SEUSS-LIKE1 (SLK1) and SLK2 have been proposed to function redundantly in diverse developmental processes; however their function in the abiotic stress response is unknown. Moreover, the molecular functions of LUH, SLK1 and SLK2 remain obscure. Here, we show the molecular function of LUH, SLK1 and SLK2 and the role of this complex in the abiotic stress response.

**Results:**

The *luh*, *slk1* and *slk2* mutant plants shows enhanced tolerance to salt and osmotic stress conditions. SLK1 and SLK2 interact physically with the LUFS domain in LUH forming SLK1-LUH and SLK2-LUH co-repressor complexes to inhibit the transcription. LUH has repressor activity, whereas SLK1 and SLK2 function as adaptors to recruit LUH, which in turn recruits histone deacetylase to the target sequences to repress transcription. The stress response genes *RD20*, *MYB2* and *NAC019* are expressed at elevated levels in the *luh*, *slk1* and *slk2* mutant plants. Furthermore, these stress response genes are associated with decreased nucleosome density and increased acetylation levels at H3K9 and H3K14 in the *luh*, *slk1* and *slk2* mutant plants.

**Conclusions:**

Our results indicate that SLK1, SLK2 and LUH form a co-repressor complex. LUH represses by means of an epigenetic process involving histone modification to facilitate the condensation of chromatin thus preventing transcription at the target genes.

## Background

Plant’s ability to perceive and respond to various environmental stresses including too little water (drought), too much salt (salinity) and extremes of temperature, depends on appropriate regulation of gene expression. Abiotic stress causes both up and down regulation of gene expression [1-3] . Many of the up regulated genes encode proteins that can be classified into two groups: genes coding for the transcription factors and genes encoding proteins involved directly in response mechanisms [4]. Genes of both classes are controlled at the level of transcription. The molecular mechanisms of specific transcriptional factors that bind to the conserved cis-acting promoter elements in plants are well studied, especially for the abiotic stress-induced up-regulated genes [4-7]. In contrast, mechanisms whereby abiotic stress regulated genes are kept silent in the absence of stress has not been well investigated. To silence gene expression, eukaryotes employ transcriptional repression as a key regulatory mechanism. Transcriptional repression plays a critical role in cell fate specification and body patterning in both animals and plants [8,9]. Transcriptional activation and repression occur within the context of chromatin organization in eukaryotes. Chromatin structure is a governed process often associated with epigenetic regulation, namely, histone post-translational modifications, histone variants and DNA methylation that alter chromatin compaction resulting in altered accessibility of genes to transcriptional regulation [10,11].

Transcriptional repression is mediated by an important and extensively studied class of co-repressors, those belonging to the Gro/Tup1 family, including Tup1 in yeast (*Saccharomyces cerevisiae*, *Schizosaccharomyces pombe*, and *Candida albicans*), Groucho (Gro) in *Drosophila*, and Transducin-like enhancer of split (TLE) in mammals. These co-repressor proteins, collectively called the Gro/Tup1 family [12,13], do not possess direct DNA-binding capability. They repress a diverse number of target genes through targeted recruitment to the DNA template via protein-protein interactions with a variety of DNA-bound transcription factors to mediate repression [8].

The Gro/Tup1 family consists of 13 members in *Arabidopsis*, and the functions of only a few have been studied [9,12,13]. LEUNIG (LUG) was the first Gro/Tup1 family member to be characterized in *Arabidopsis*[12]. The LUG protein has LisH and LUFS domains at the N-terminus, and resembles Gro/Tup1 in having a Q-rich and seven WD domains. The LisH (lissencephaly homology) domain is a dimerization motif that is present in all plant Gro/Tup1 proteins. The LUFS (named after *L*UG, L*U*H, yeast *F*lo8 and human *S*SDP) domain is present only in LUG and LUH among the Gro/Tup1 family members present in *Arabidopsis*. LUFS domain is involved in protein-protein interactions [12,13]. The LUFS domain in LUG interacts physically with SEUSS (SEU), a Q rich protein with a conserved domain that is similar to the dimerization domain of the LIM-domain binding (Ldb) family of transcriptional co-regulators in mouse and *Drosophila*[14,15]. SEU forms a co-repressor complex with LUG and acts as an adapter between LUG and a variety of transcription factors to mediate repression of diverse target genes during floral organ identity, floral patterning and abaxial organ identity in leaves [16-18].

LUH is another member of the Gro/Tup1 family and highly similar to LUG with 44% identity in *Arabidopsis*[12,13,19]. Thus, not surprisingly LUH functions redundantly to some extent with LUG in abaxial organ identity in leaves and identity of floral organs [18,19]. Furthermore, LUH interacts with SEU in a yeast two hybrid assay suggesting that SEU-LUH complex could functionally substitute for the SEU-LUG complex to mediate repression at target gene which may possibly explain the redundant functions [19]. In addition, LUH also interacts with SLK1 and SLK2 and functions redundantly with LUG in abaxial organ identity [18]. Until recently, LUH function in addition to its minor role in development was not known. Several recent reports indicate that LUH plays an important role in regulating pectin structure and mutants lacking LUH fail to release mucilage from the seed coat. One relevant target of LUH is *MUM2*, a β-galactosidase involved in the modification of the mucilage [20-22]. At present, there are two plausible mechanisms to account for *MUM2* regulation. The first is by LUH acting as a direct positive regulator of *MUM2*. The other mechanism involves LUH acting as negative regulator of a *MUM2* repressor.

Although LUH shows significant sequence similarity with LUG, the molecular function of LUH remains unclear. The only known function of LUH is its major role in mucilage secretion in *Arabidopsis*. In this study, we present results indicating involvement of LUH in the abiotic stress response. We demonstrate that LUH functions as a transcriptional repressor similar to Gro/Tup1 family proteins. Additionally, we show that the conserved LUFS domain in LUH physically interacts with adaptor proteins SLK1 and SLK2 which do not show repressor activity themselves. The *luh*, *slk1* and *slk2* mutant plants shows elevated salt and osmotic stress tolerance and higher expression levels of abiotic stress responsive gene under non-stress conditions. In addition, LUH physically interacts with histone H2B and H3 and either directly or indirectly regulates chromatin structure at the abiotic stress responsive genes. These data provide an insight into the novel roles for LUH, SLK1 and SLK2 in abiotic stress response gene regulation and illuminate LUH function in chromatin remodeling.

## Results

### *luh-4*, *slk1-1* and *slk2-1* plants exhibit tolerance to salt and osmotic stress

**Figure 1:**
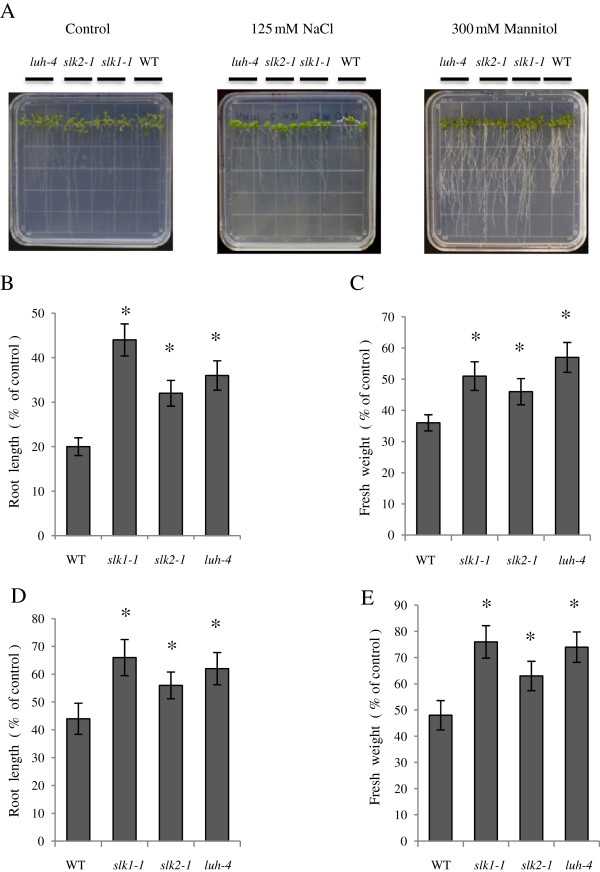
**The *luh-4, slk1-1 *and *slk2-1 *mutants show tolerance to salt and osmotic stress. (A)** The single mutants and control plants were grown on the MS medium for six days. The plants were transferred to MS medium as a control and MS medium supplemented with 125 mM NaCl and 300 mM mannitol for salt and osmotic stress treatment respectively. The plants were grown in growth chamber and photographed after 10 days for control, 15 days for salt and 25 days for osmotic treatment plates. **(B)** Root length of plants grown on the MS medium with 125 mM NaCl for 15 days. **(C)** Fresh weight of plants grown on the MS medium with 125 mM NaCl for 15 days. **(D)**  Root length of plants grown on the MS medium with 300 mM mannitol for 25 days. **(E)** Fresh weight of plants grown on the MS medium with 300 mM mannitol for 25 days. The root length and fresh weight for salt and osmotic stress is presented as a percentage relative to plants grown on MS medium without stress treatment. Error bars are SE with 20 – 25 plants per replicate (n = 4). Asterisks indicate values that are significantly different from the wild type plants (*P <0.05, Student’s t test).

Comparison of expression profiles between LUG and LUH revealed that both the genes are expressed at comparable levels in all tissues under normal condition. Interestingly, LUH expression level is elevated in both biotic and abiotic stress in contrast to LUG which remained unchanged or reduced [19]. Since LUH expression is enhanced in abiotic stress and interacts with SEU [19], we sought to determine whether the LUH-SEU complex plays a role in abiotic stress. We subjected *luh-4* and *seu-1* plants to salt and osmotic stress. Plants with mutation in SEU showed unchanged tolerance to salt and osmotic stress (Additional file 1: Table S1) that could be attributed to the functional redundancy within the SEU family proteins. *Arabidopsis* encodes three SEU-like proteins (SLK1, SLK2 and SLK3) and these proteins function redundantly with SEU in flower development [23]. We hypothesized that SLK may be involved in the abiotic stress and functions redundantly with SEU in flower development, because *slk1-1* and *slk2-1* single and double mutants do not show any defect in flower development. To test this, loss of function mutants *slk1-1*, *slk2-1* and *luh-4* were examined for altered response to salt and osmotic stress. We observed difference in the root lengths in the single mutants compared to wild type plants when grown on MS medium supplemented with 125 mM NaCl for salt stress and 300 mM mannitol for osmotic stress (Figure 1A). The root length was longer in the single mutants compared to the wild type plants when grown on MS medium containing 125 mM NaCl and 300 mM mannitol (Figure 1B, D). In addition, the fresh weight of the single mutants were higher compared to the wild type plants when grown on MS medium supplemented with 125 mM NaCl and 300 mM mannitol (Figure 1C, E). Interestingly, on MS medium without stress treatment, root length of *luh-4* and *slk1-1* mutants was slightly shorter compared to wild type plants due to slower root growth. The differences in the root length between *luh-4*, *slk1-1* mutants and wild type plants became negligible with longer periods of incubation on MS medium. The fresh weight of *slk1-1*, *slk2-1*, and *luh-4* mutants were comparable to wild type plants when grown on MS medium without stress treatment.

To verify whether the salt and osmotic stress tolerance in *slk1-1*, *slk2-1* and *luh-4* is due to loss of function. We transformed the mutants with native gene promoter containing wild type coding sequence. Transgenic mutants with wild type coding sequence complemented the salt tolerance phenotype and were similar to wild type plants when grown on MS medium supplemented with 125 mM NaCl for salt stress (Additional file 2: Figure S1A). The root length of complemented mutants were comparable to the wild type plants when grown on medium containing 125 mM NaCl and 300 mM mannitol (Additional file 2: Figure S1B, D). In addition, the fresh weight of the complemented mutants were similar to the wild type plants when grown on medium supplemented with 125 mM NaCl and 300 mM mannitol (Additional file 2: Figure S1C, E). Expression level of *SLK1*, *SLK2* and *LUH* gene in the complemented plants were similar to their expression in the wild type plants (Additional file 3: Figure S2).

To determine whether the double mutants show enhanced tolerance to the salt and osmotic stress compared to the single mutants, we constructed *slk1-1 /luh-4* and *slk2-1/luh-4* double mutants. The double mutants did not exhibit significant differences in the root length and fresh weight compared to the single mutants when subjected to salt and osmotic stress (Additional file 1: Table S1). Additionally, altered response to the plant hormone abscisic acid (ABA up to 10 μM concentration) and freezing tolerance (-4°C to -10°C) was tested and these responses were unchanged in the single mutants compared to the wild type plants (unpublished data). Collectively these data show that loss of function in LUH, SLK1 and SLK2 results in enhanced tolerance to salt and osmotic stress in the single mutants compared to the wild type plants.

### SLK1 and SLK2 interact with the LUFS domain in LUH

**Figure 2:**
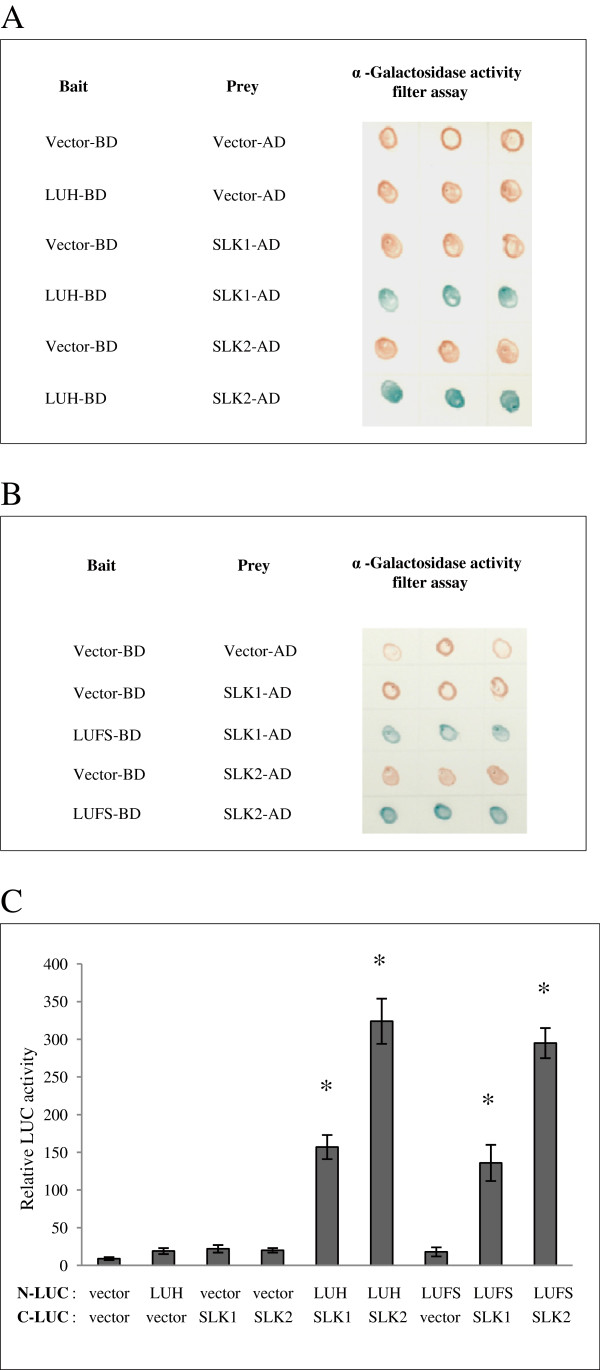
**SLK1 and SLK2 interact with LUFS domain of LUH in Yeast Two Hybrid and in *planta*. (A)** LUH was expressed from bait vector as BD fusion and SLK1 or SLK2 from prey vector as AD fusion. Results of yeast two hybrid assay indicating the activation of α- Galactosidase reporter expression. Blue color indicates positive interaction. **(B)** The LUFS domain of LUH (amino acid residues 1-88) was expressed as BD fusion and SLK1 or SLK2 as AD fusion that is sufficient for interaction with SLK1 and SLK2. **(C)***Arabidopsis* protoplasts were transfected with 15 μg of each plasmid containing LUH, LUFS domain as N-LUC fusion and SLK1 or SLK2 as C-LUC fusion. *CaMV 35S::Renilla LUC *reporter was used as an internal control for transfection. *LUC* activity is expressed relative to the *R-LUC* activity. Error bars are SE (*n* = 3). Asterisks indicate values that are significantly different from the vector control (*P <0.05, Student’s t test).

It has been shown that LUH interacts with SLK1, SLK2 and SLK3 in yeast two hybrid assay [18]. Our yeast two hybrid assay also showed interaction between LUH fused with the Gal4 DNA binding domain (BD) and SLK1 and SLK2 fused with the Gal4 activation domain (AD) (Figure 2A). We have previously shown that SEU interact with the LUFS domain in LUG, thus raising the question whether SLK1 and SLK2, interact with the LUFS domain in LUH [14]. Indeed, yeast two hybrid analysis indicated that the LUFS domain in LUH is sufficient for physical interaction with SLK1 and SLK2 (Figure 2B).

To confirm LUH, SLK1 and SLK2 interactions in plants, we performed split *luciferase* complementation assays in *Arabidopsis* protoplasts [24] by fusing *luciferase* N-terminal fragment translationally to full length LUH and to the LUFS domain alone. The C-terminal fragment was fused to SLK1 or SLK2. In agreement with the yeast two hybrid assays, protoplasts transfected with *LUH-SLK1* and *LUH-SLK2* plasmids showed elevated levels of *luciferase* activity compared to vector treated and N-terminal fragment fused with LUH alone (Figure 2C). The SLK2 interaction with LUH was higher compared to SLK1. Moreover, SLK1 and SLK2 interaction with the LUFS domain were as strong as full length LUH supporting the idea that the LUFS domain is sufficient for interaction with SLK1 and SLK2 to form LUH-SLK1 and LUH-SLK2 co-repressor complexes *in vivo* (Figure 2C).

### LUH has repressor activity

**Figure 3:**
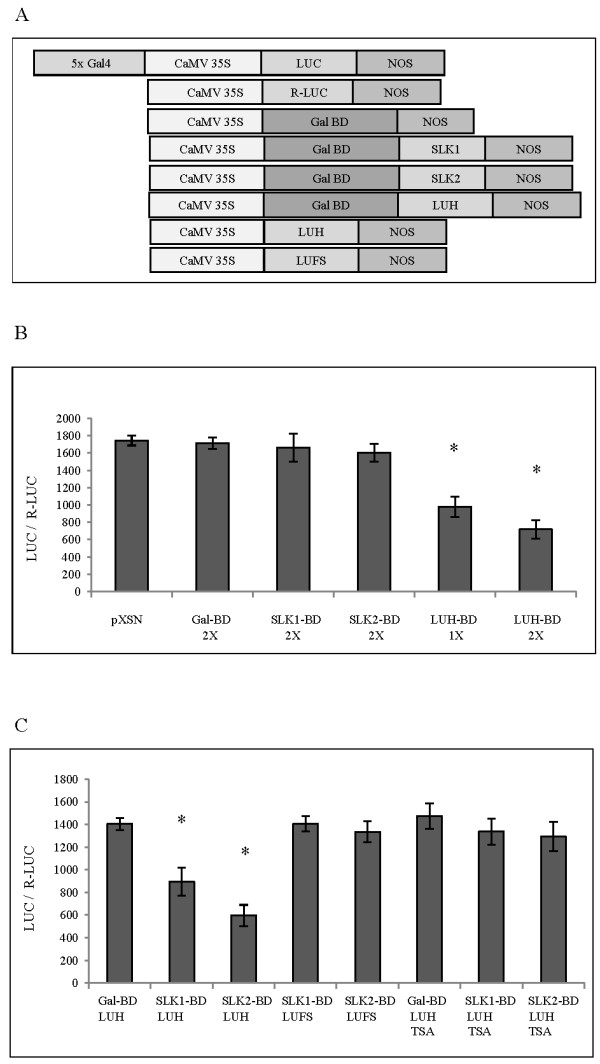
**LUH has repressor activity and SLK1, SLK2 acts as adapter protein for LUH recruitment. (A)** Schemes of the reporter and effector constructs. In the reporter plasmid 5X Gal4 binding site were fused to *CaMV 35S* promoter and *LUC* gene. *Renilla luciferase* (*R-LUC*) reporter is used as an internal control for transfection. In the effector plasmid, *SLK1*, *SLK2* and *LUH* gene were fused to the *Gal4-BD* under the control of *CaMV 35S* promoter. For effector *LUH* and *LUFS* without *Gal4-BD*, the gene was fused to *CaMV 35S* promoter and noplaine synthase terminator. **(B)***Arabidopsis* protoplasts were transfected with *luciferase* reporter construct (10 microgram DNA) plus *Renilla luciferase* (0.5 microgram DNA) together with the effector construct. The ratios of *LUC/R-LUC* indicate relative reporter activity. Twenty microgram of pXSN (vector), *Gal-BD*, *SLK1-BD*, *SLK2-BD* and *LUH-BD* DNA was used for 2X and ten microgram for 1X in repression assay. **(C)** To determine adapter role of SLK1 and SLK2, the *Arabidopsis* protoplasts were transfected with reporters as above together with ten microgram of each *Gal-BD, SLK1-BD*, *SLK2-BD* DNA and 20 microgram of *CaMV 35S::LUH or CaMV 35S::LUFS* DNA. The ratios of *LUC/R-LUC* indicate relative reporter activity. 20 μM TSA was used to inhibit HDAC activity in protoplasts. Error bars are SE (*n* = 3). Asterisks indicate values that are significantly different from the pXSN vector control for **(B)** and *Gal-BD* for **(C)** (*P <0.05, Student’s t test).

To determine whether LUH, SLK1 and SLK2 could function as transcriptional repressors, a repression assay in *Arabidopsis* protoplasts was performed. The Gal4 DNA binding domain was fused with *SLK1*, *SLK2* and *LUH.* The constructs were co-transfected into *Arabidopsis* protoplasts along with a reporter construct *5XUAS*_*Gal4*_*CaMV 35S::LUC* containing 5x Gal4 binding sites upstream of *CaMV 35S* constitutive promoter and the effect on *luciferase* expression was quantitated (Figure 3A). Transfection with *SLK1-BD* and *SLK2-BD* alone did not affect the reporter gene expression. In contrast, *LUH-BD* significantly reduced reporter gene expression in a concentration dependent manner, indicating that LUH functions as a transcriptional repressor *in vivo* (Figure 3B). To explore the possibility that SLK1 and SLK2 may serve as adaptor proteins to aid the interaction between LUH and DNA-binding transcription factors, as seen for the SEU-LUG complex [17], *SLK1-BD* or *SLK2-BD* DNA was co-transfected with the *CaMV 35S::LUH* or *CaMV 35S::LUFS* construct, and effects on reporter expression were quantitated [14]. These results revealed that in the presence of LUH, SLK1 or SLK2 significantly reduced reporter gene expression (Figure 3C). In contrast, LUFS did not reduce the reporter expression, suggesting that the Q-rich and WD domain in the LUH is required for the repressor activity (Figure 3C). These data indicate that LUH has repressor function and confirms the hypothesis that SLK1 and SLK2 function as adapter proteins to recruit LUH to the promoter to inhibit gene transcription*.*

Co-repressors in the Gro/Tup1 family mediate repression by recruiting histone deacetylases (HDACs) to the target genes [12,13]. In order to determine whether a similar mechanism is utilized by LUH, we performed repression assays in protoplasts in presence of TSA, an inhibitor of HDAC activity. Interestingly, LUH failed to repress the reporter gene in the presence of TSA (Figure 3C) indicating that LUH employs a highly conserved process that involves recruitment of HDACs to mediate repression at the target loci [8].

**Figure 4:**
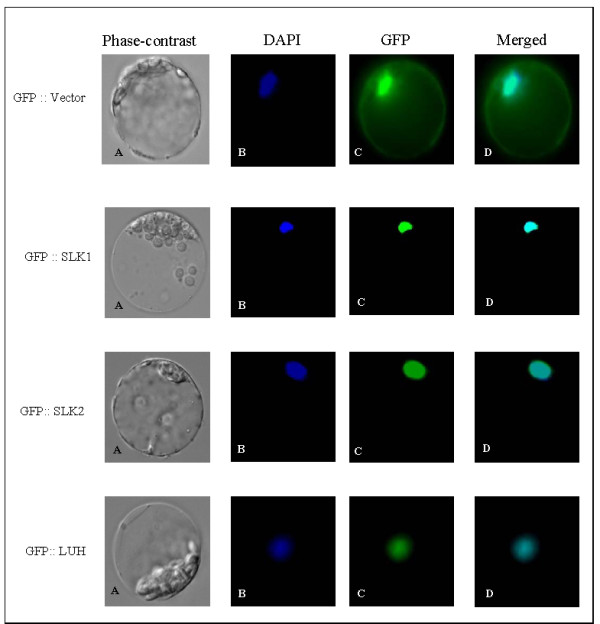
**Cellular localization of SLK1, SLK2 and LUH. (A)** Phase-contrast image of the protoplast. **(B)** Protoplast image stained with DAPI for nuclear localization. **(C)** Protoplast image with GFP localization. **(D)** Protoplast image merged with DAPI and GFP localization. The images were captured using 60X objective lens.

Since SLK1, SLK2 and LUH form a co-repressor complex and SLK1 and SLK2 have typical nuclear localization signal (NLS) in contrast to LUH that has atypical NLS, we investigated subcellular localization by fusing with GFP and expressing the fusion proteins in *Arabidopsis* mesophyll protoplasts. As expected, results from fluorescent microscopy indicated that the SLK1, SLK2 and LUH are nuclear localized (Figure 4). Taken together, these results reveal that SLK1, SLK2 and LUH are present in the nucleus, supporting the idea that they form co-repressor complexes and mediate repression by recruiting HDAC to the target genes.

### LUH negatively regulates abiotic stress response genes

**Figure 5:**
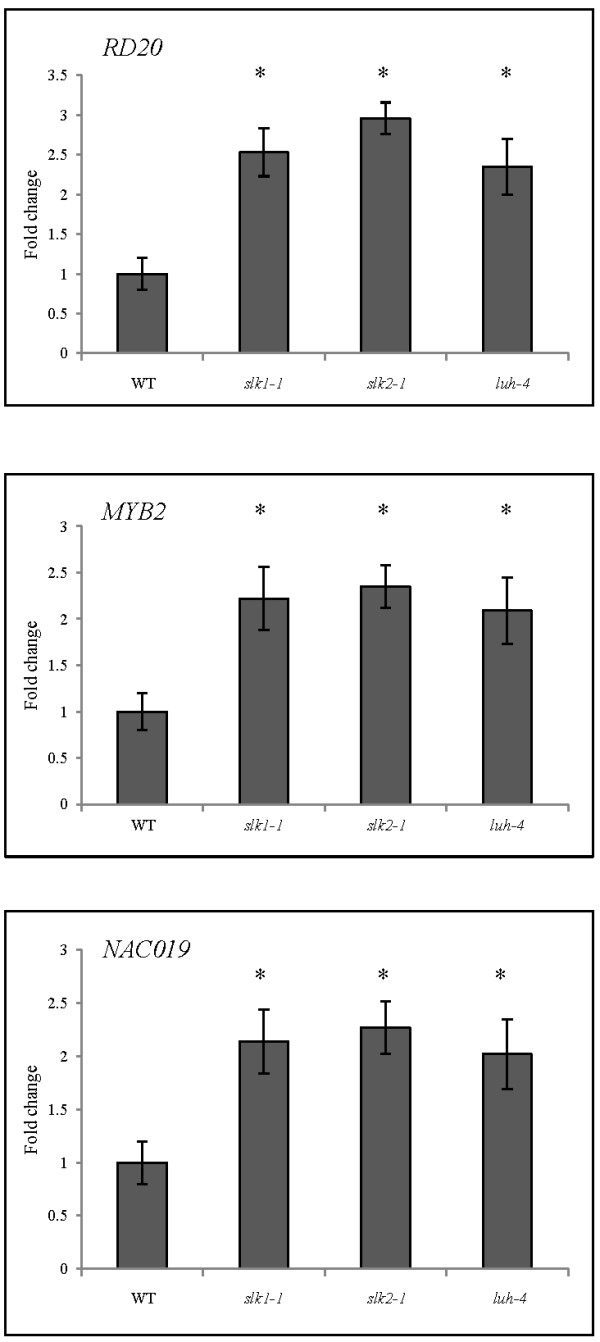
**Analysis of stress responsive gene expression in *slk1-1*, *slk2-1 *and *luh-4 *mutants.** Transcript levels of *RD20*, *MYB2* and *NAC019* were quantitated using qRT-PCR in the wild type and mutant plants with *ACTIN2* as an internal control. Data are depicted as fold change obtained from three biological replicates. Error bars are SE (*n* = 3). Asterisks indicate values that are significantly different from the wild type plants (*P <0.05, Student’s t test).

Involvement of SLK1, SLK2 and LUH in salt and osmotic tolerance indicated that abiotic stress response gene expression is altered in these mutants thereby conferring tolerance to the abiotic stress. To identify the genes that are differentially expressed in *slk1-1*, *slk2-1* and *luh-4* mutants compared to wild type plants, we performed quantitative RT-PCR for some well-known genes that confer abiotic stress tolerance [7,25] and compared their expression to *ACTIN2* gene as an internal control. Elevated expression of *RD20*, *MYB2* and *NAC019* transcripts was observed in the *slk1-1*, *slk2-1* and *luh-4* mutants compared to wild type plants under non stress conditions (Figure 5). In contrast, expression level of *RD20*, *MYB2* and *NAC019* transcripts were comparable to wild type in the complemented plants (Additional file 4: Figure S3).

MYB2 and NAC019 are transcription factors that are implicated in the regulation of several abiotic stress response genes [26-28]. Furthermore, elevated expression of *RD20* confers abiotic stress tolerance [29]. These data indicate that the loss of function in SLK1, SLK2 and LUH results in increased expression of *RD20*, transcription factors MYB2 and NAC019 and could possibly result in the improved tolerance to the abiotic stress in these mutant plants.

### LUH alters the chromatin state at the abiotic stress response genes

**Figure 6:**
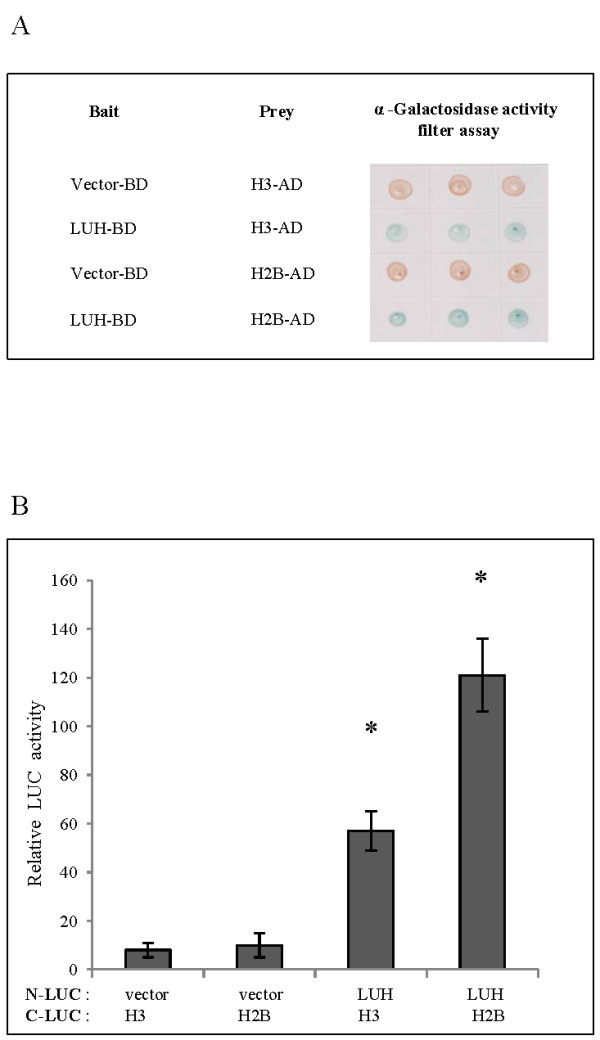
**Histone H3 and H2B interacts with LUH in Yeast Two Hybrid and in *planta*. (A)** LUH was expressed from bait vector as BD fusion and H3 or H2B from prey vector as AD fusion. Results of yeast two hybrid assay indicating the activation of α- Galactosidase reporter expression. Blue color indicates positive interaction. **(B)***Arabidopsis* protoplasts were transfected with 15 μg of each plasmid containing LUH as N-LUC fusion and H3 or H2B as C-LUC fusion. *CaMV 35S::Renilla LUC* reporter was used as an internal control for transfection and *LUC* activity is expressed relative to the *R-LUC* activity. Error bars are SE (*n* = 3). Asterisks indicate values that are significantly different from the vector control (*P <0.05, Student’s t test).

A number of studies indicate that the Gro/Tup1 family of co-repressors present in the repressor complex interacts with histones to regulate gene activity. In yeast, Tup1 interacts with histone H3 and H4, and human TBL1 interacts with histone H4 and H2B [30,31]. Yeast two hybrid assays revealed that LUH interacts in a similar manner with the histone H2B and H3 (Figure 6A). We confirmed this interaction quantitatively using split *luciferase* complementation assays in *Arabidopsis* protoplasts. The results show that LUH interaction with histone H2B is higher compared to histone H3 (Figure 6B). Differences in the histone interaction between LUH, TBL1 and Tup1 are not surprising taking into consideration the disparity between these co-repressors at the N-terminal sequences.

**Figure 7:**
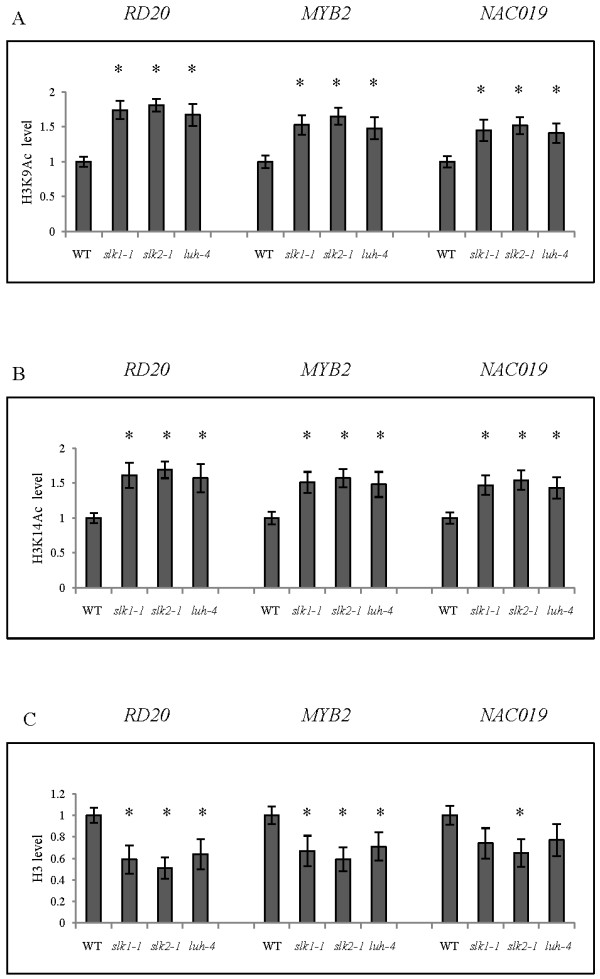
**Alterations in the acetylation levels and nucleosome density on *RD20*, *MYB2 *and *NAC019 *genes in the *slk1-1*, *slk2-1 *and *luh-4 *mutants. (A)** and **(B)** Relative acetylation level at H3K9 and H3K14 position determined by ChIP assays using specific antibodies and normalized to internal control *ACTIN7* gene. **(C)** Changes in the nucleosome density were determined by ChIP assays using anti-histone H3 C-terminal antibodies and normalized to internal control *ACTIN7* gene. The value of wild type plants was arbitrarily given as 1. The data are average of three biological replicates. Error bars are SE (*n* = 3). Asterisks indicate values that are significantly different from the wild type plants (*P <0.05, Student’s t test).

Since LUH interacts with histone H3 and H2B and requires HDAC for the repressor activity, we examined the histone acetylation level at the target genes that showed elevated expression in the *slk1-1*, *slk2-1* and *luh-4* mutants compared to the wild type plants. It is well established that the histone H3 N-terminal tail is acetylated at Lys-9, Lys-14, Lys-18 and Lys-23 positions and these modifications are required for promoting active transcription [32,33]. Therefore, we performed ChIP assays at the first exon of coding region in *RD20*, *MYB2* and *NAC019* gene for the histone H3 acetylation at Lys-9 and Lys-14 positions with respective antibodies (The position and size of the fragments are shown in Additional file 5: Figure S4). Our data shows increased histone H3 acetylation level at the first exon in both Lys residues in the *slk1-1*, *slk2-1* and *luh-4* mutants compared to wild type plants (Figure 7A, B). We also examined nucleosome density by ChIP assay at the first exon of coding region with histone H3 C-terminal antibodies to determine changes in histone H3 levels in the *slk1-1*, *slk2-1* and *luh-4* mutants compared to wild type plants in *RD20*, *MYB2* and *NAC019* gene. We found decreased histone H3 levels at the first exon of coding region in *RD20*, *MYB2* and *NAC019* gene in the *slk1-1*, *slk2-1* and *luh-4* mutants compared to wild type plants (Figure 7C). These results are consistent with the understanding that the active gene transcription is associated with reduced nucleosome density [34].

We did not determine the acetylation status in H2B due to the lack of plant specific antibodies. In conclusion, LUH interacts with histone H2B and H3 and recruits HDAC to eliminate the acetylation on histone H3 at the positions Lys-9 and Lys-14. Furthermore, the presence of LUH could increase the nucleosome density resulting in the condensation of the chromatin and hindering the active transcription at the target genes.

## Discussion

In *Arabidopsis*, LUG and TOPLESS (TPL) are the most studied Gro/Tup1 co-repressors that are implicated in developmental processes and hormone signaling [9,12,13]. LUH is the homolog of LUG and plays critical role in mucilage excretion [20-22]. Expression profile analysis indicated that the LUH is differentially regulated during abiotic stress compared to LUG and could play a role in the abiotic stress response [19]. Surprisingly, HOS15, belonging to Gro/Tup1 family, was identified in a forward genetic screen involving abiotic stress response, and loss of function in HOS15 results in freezing sensitivity [35]. These studies prompted us to investigate LUH function in abiotic stress response and here we show that LUH is indeed involved in abiotic stress response thus broadening the function of LUH. Loss of function mutation in LUH results in plants that are more tolerant to salt and osmotic stress compared to the wild type plants. LUH interacts with SEU, an adaptor protein that links LUH to the transcription factor, and interestingly, SEU mutants do not show tolerance to salt and osmotic stress. In *Arabidopsis*, there are three SEU like genes [23], and we found that loss of function in SLK1 and SLK2 confers salt and osmotic tolerance similar to LUH when mutant plants were subjected to the stress conditions. SEU, SLK1 and SLK2 function redundantly in embryonic development mediated through plant hormone auxin and in the outer integument development in the ovule [23]. Our results provide a novel function for the SLK1 and SLK2 in the abiotic stress response outside their role in the development. Double mutant analysis with *slk1-1/luh-4* and *slk2-1/luh-4* for salt and osmotic stress indicated that *slk1*, *slk2* and *luh* functions in the same genetic pathway. We did not observe differential responses in *slk1*, *slk2* and *luh* compared to wild type plants during freezing and plant hormone ABA treatment.

Genetic analysis indicated that LUH function is dependent on SLK1 and SLK2. Yeast two hybrid and in *planta* analyses in protoplasts indicated that the LUH interacts with SLK1 and SLK2 confirming an earlier study [18]. Interestingly, LUFS domain in LUH is sufficient for interaction with SLK1 and SLK2 which is similar to the interaction of SEU with the LUFS domain in LUG [14]. SEU, SLK1 and SLK2 are highly similar with a centrally positioned Q-rich region containing a Ldb1/chip conserved domain (LCCD) that is likely to interact with the LUFS domain [23]. Phylogenetic analysis indicated that SEU is more closely related to SLK2 than to SLK1 which could explain the stronger interaction between LUH and SLK2 compared to SLK1 [18,23].

The molecular functions of SLK1, SLK2 and LUH were unknown. Our results indicate that only LUH has transcriptional repressor activity. Interestingly, co-transfection with either *SLK1-BD* or *SLK2-BD* with *CaMV 35S::LUH* gave repressor activity. These results indicate that SLK1 and SLK2 function as adaptors to recruit LUH, which serves as the repressor within this complex. A recent study showed that LUH functions as an activator in *Arabidopsis* protoplasts in contrast to the repressor function observed in our study [21]. One possibility for this observed function of LUH could be due to the different reporter systems used in the protoplast assay. In our assay, the reporter has 5X Gal sequence upstream of constitutive *CaMV 35S* promoter; in contrast, the published study used 4X Gal sequence with the reporter gene without constitutive promoter in between 4X Gal sequence and the reporter gene [21]. In our view, LUH functions as the repressor and this is supported by the observation that in the *luh* mutant, expression of LUG which has repressor activity [14,36] restores the mucilage deficiency phenotype in the *luh* plants [20]. It is possible that in some cellular or developmental contexts LUH may function as an activator, although this mode of regulation has little empirical support.

The repressor activity of the LUH in protoplasts is eliminated by addition of TSA suggesting that the repression is mediated by recruiting HDAC. *Arabidopsis* encodes 18 HDACs and plays critical role in development, growth and hormone signaling [37-40]. Recent studies indicate that HDA19 genetically and physically interacts with co-repressors LUG and TPL, and has been implicated in flower development [9,36,41]. Our preliminary results indicate that LUH does not interact with the HDA19 and the identity of the HDAC that interacts with the LUH remains to be established (unpublished data).

To explain the observed salt and osmotic stress tolerance in *slk1*, *slk2* and *luh* mutants, we performed quantitative RT-PCR for the abiotic stress response genes that show elevated transcripts level compared to the wild type plants thus contributing to the stress tolerance. Our results indicate that *RD20*, *MYB2* and *NAC019* genes are expressed at elevated level in these mutants compared to wild type plants. *RD20* gene is a well known abiotic stress inducible marker and participates in stomatal control and transpiration in *Arabidopsis* thus conferring abiotic stress tolerance [29]. The *MYB2* gene encodes a R2R3 MYB domain-containing transcription factor that regulates several salt and drought stress responsive genes [27,42]. NAC domain-containing transcription factors are prominent plant specific transcription factors and *NAC019* is one of the 110 genes that are encoded in the *Arabidopsis* genome [28]. *NAC019* gene is induced by salt and dehydration stress, and over expression in the transgenic plants results in the induction of several stress response genes hence conferring abiotic stress tolerance [38,43,44]. Interestingly, *NAC019* regulatory region has an MYB binding site, and MYB2 transcription factor binds to the *NAC019* regulatory region in a yeast one hybrid assay [43]. However, *NAC019* gene activation by MYB2 in *planta* has not been demonstrated.

The three identified target genes are not adequate to explain the observed salt and osmotic stress phenotype in the *luh-4*, *slk1-1* and *slk2-1* mutant plants. Therefore, it appears that several positive factor genes are expressed in these mutants compared to wild type plants that confer abiotic tolerance. Further studies are required to identify additional target genes.

Since SLK1, SLK2 and LUH lack DNA binding domain, the mechanisms of recruitment of SLK1-LUH and SLK2-LUH complexes to the regulatory region of *RD20*, *MYB2* and *NAC019* genes are unknown. Among the possible mechanisms, one could be that SLK1 and SLK2 interacts with different sequence specific transcription factor or SLK1 and SLK2 form heterodimeric complexes that bridge the transcription factor and LUH at the target regulatory region. Identification of specific transcription factors that interact with SLK1 and SLK2 and *in vivo* association at the regulatory region would illustrate the precise mechanism of SLK1-LUH and SLK2-LUH recruitment to the target genes.

Chromatin structure within a gene largely determines its transcriptional state and expression levels and can be changed with modification at the N- terminal tails of histones. One of the key mechanisms in chromatin remodeling is histone acetylation and deacetylation, mediated by the enzymes histone acetyl transferase (HAT) and HDAC respectively [33,39]. The role of chromatin remodeling is well established in transcriptional gene silencing and in control of flowering response by vernilization in *Arabidopsis*[10,45]. Recent studies suggest that abiotic stress response gene expression also depends on chromatin remodeling, yet how this distinctive chromatin state is established is not known [46]. In addition, the function of Gro/Tup1 family of co-repressors in chromatin remodeling in *Arabidopsis* is not well understood. Our results demonstrate that LUH interacts with histone H3 and H2B. Furthermore, the chromatin state is altered at target genes that are expressed at elevated levels. We observed that nucleosome density at target genes *RD20*, *MYB2* and *NAC019* that are expressed in the *slk1-1*, *slk2-1* and *luh-4* mutants are reduced compared to the wild type plants. These results are consistent with the observation that higher nucleosome density within a gene inhibits transcription by limiting RNA polymerase processivity [34]. In plants, histone H3 modification at positions Lys-9 and Lys-14 is positively correlated with gene activation, and the deacetylated status with inactive transcription [33]. Our results indicate that histone H3 is acetylated at positions Lys-9 and Lys-14 on the target genes *RD20*, *MYB2* and *NAC019* that are highly expressed in the *slk1-1*, *slk2-1* and *luh-4* mutants compared to the wild type plants. These data indicate that LUH prevents the expression of target genes by recruiting HDACs that deacetylate histone H3 at positions Lys-9 and Lys-14. Further studies are needed to establish the presence of LUH, SLK1 and SLK2 at the regulatory sequence of the target genes to modify the chromatin status.

LUH is induced during abiotic stress in contrast to LUG suggesting that LUH plays an important role in abiotic stress response. Interestingly SLK1 and SLK2 are induced in response to osmotic stress (unpublished data). There are several possible roles that LUH can participate in regulating abiotic stress response in plants. First, during abiotic stress several genes are induced that confer tolerance to the abiotic stress and increased LUH expression could form complex with SLK1/SLK2 and negatively regulate genes that are detrimental to the abiotic stress tolerance. Second, one of the main mechanisms that plants employ to endure abiotic stress is by reprogramming the developmental pathway so that important growth phases that are sensitive to abiotic stress are delayed [40]. The LUH-SLK1 and LUH-SLK2 complexes could repress the genes that are involved in the transition of growth phase. Third, LUH-SLK1 and LUH-SLK2 complexes could regulate the abiotic stress pathway by controlling the length or level of response by regulating the positive or negative determinant genes by negative feedback loop.

## Conclusions

SLK1 and SLK2 function as adapters to form SLK1-LUH and SLK2-LUH complexes with LUH possessing repressor activity. How the SLK1-LUH and SLK2-LUH complexes are recruited to the promoter of the abiotic stress response genes remains to be determined. LUH could exert its repressive effect on the target genes by recruiting histone deacetylase that facilitates deacetylation of histone H3 associated with promoter of target genes. The binding of the LUH to the histone H3 results in the condensation of chromatin and increased nucleosome density, thus preventing gene transcription by RNA polymerase at the target genes. Further studies are needed to determine the transcription factors that interact with SLK1 and SLK2 to recruit LUH to the regulatory sequence of target genes. Microarray analysis in *slk1*, *slk2* and *luh* mutant plants will provide additional insight into the abiotic stress response genes regulated by SLK1, SLK2 and LUH.

## Methods

### Plant materials and abiotic stress treatment conditions

The Arabidopsis (*Arabidopsis thaliana*) ecotype Columbia (Col0) and Landsberg *erecta* (L*er*) was used as wild type controls. *luh-3* (seed stock no. SALK_107245C), *luh-4* (seed stock no. SALK_097509), *slk1-1* (seed stock no. CS65896), *slk2-1* (seed stock no. CS65894) mutant lines were obtained from the Arabidopsis Biological Resource Center (ABRC). All the mutant lines are in the Col0 background except for *seu-1* which is in L*er* background.

The wild type and mutant seeds were sterilized with 50% bleach and planted on half-strength Murashige and Skoog salt, 1% sucrose, 0.8% agar (MS) media and incubated at 22°C under long-day light conditions in the growth chamber (Percival). For abiotic stress treatment, six day old seedlings were transferred to the MS media with or without 125 mM NaCl or 300 mM mannitol and incubated in the growth chamber under long-day conditions at 22°C. Root length and fresh weight are expressed as a percentage relative to plants grown on MS medium without stress treatment after 15 days for salt and 25 days for mannitol treatment.

### Yeast Two hybrid assay

*LUH* (G12254), *SLK1* (G66746) and *SLK2* (G10219) cDNA clones were obtained from Arabidopsis Biological Resource Center. The cDNA clones were amplified by PCR with Pfu ultra (Agilent Technologies) and cloned in frame by In-Fusion HD Cloning Plus (Clontech) into vector pGBKT7 (Clontech) at *Nde1*-*Sal1* and pGADT7 (Clontech) at *Nde1*-*BamH1* sites to generate Gal4-BD and Gal4-AD fusions respectively. The LUFS domain (1-88 amino acids) was PCR amplified from *LUH* cDNA and cloned in frame by In-Fusion HD Cloning Plus into vector pGBKT7 at *Nde1*-*Sal1* site. The histone gene *H3* (AT5G65360), *H4* (AT5G59690), *H2A* (AT5G54640) and *H2B* (AT1G07790) were amplified from total RNA by RT-PCR with respective primers and inserted into vector pGADT7 at *Nde1*-*BamH1*site by In-Fusion HD Cloning Plus to generate Gal4-AD fusion. All the sequences were verified by sequencing. The yeast two hybrid interaction assays were performed in Y2H Gold (Clontech) yeast strain according to manufacturer’s protocol and reference 14. The primer sequences are listed in Additional file 6: Table S2.

### Protoplast isolation

The protoplast isolation and transfection was performed as described in [47].

### Repression assay in protoplasts

To construct reporter gene for repression assay, 342 bp *CaMV 35S* promoter was PCR amplified from pMDC32 vector [48] using primers CaMV_pUASluc2F, CaMV_pUASluc2R and inserted at *Hind111*-*EcoR1* site by In-Fusion HD Cloning Plus in the plasmid pUAS-luc2 (Addgene, plasmid: 24343) [49] to generate *CaMV 35S::LUC* vector. The *5XUAS* region was PCR amplified from pUAS-luc2 plasmid using primers 5xGal4DBF, 5xGal4DBR and inserted at *Bgl11* site in the *CaMV 35S::LUC* to generate *5XUAS*_*GAL4*_*CaMV 35S::LUC* reporter construct. The 800 bp *CaMV 35S* promoter *was* PCR amplified from pMDC32 vector and inserted at *EcoR1*-*Pst1* site by In-Fusion HD Cloning Plus in the plasmid pRL-null (Promega) to generate CaMV *35S::Renilla LUC* reporter construct. For the effector constructs, the respective gene from pGBKT7 were PCR amplified using primers pGBTK_GAL4F, pGBTK_GAL4R and cloned at *BamH1* site in the vector pXSN [50] using In-Fusion HD Cloning Plus. To generate CaMV *35S::LUH* and CaMV *35S::LUFS*, the respective genes were PCR amplified and inserted at the *BamH1* site by In-Fusion HD Cloning Plus in the pXSN vector. The protoplast transfection, reporter gene assay and trichostatin-A (TSA) treatment was performed as described in [14]. The primer sequences are listed in Additional file 6: Table S2.

### Split luciferase complementation assay

The cDNA was amplified with PCR with respective gene specific primers and inserted at *Kpn1*-*Sal1* site in the *CaMV 35S::Nluc* or *Kpn1*-*Pst1*site in the *CaMV 35S::Cluc* vector to generate N-luciferase and C- luciferase fusion respectively [24]. The transfection was performed with 5 × 10^4^ protoplasts, 15 μg of each fusion construct and 0.5 μg *CaMV 35S::Renilla LUC* as an internal control for transfection. The protoplasts were incubated in the dark for 16 h at room temperature and the luciferase assay was performed with dual luciferase reporter assay kit (Promega) and TD-20/20 luminometer (Turner Biosystems). The primer sequences are listed in Additional file 6: Table S2.

### Subcellular localization of LUH, SLK1 and SLK2

The cDNAs were amplified with respective gene specific primers and cloned into *BamH1* site by In-Fusion HD Cloning Plus in the plasmid pXDG [50] to generate GFP fusion driven by *CaMV 35S* promoter. The protoplasts were transfected with 15 μg of each plasmid DNA and incubated in the dark for 16 h at room temperature. The protoplasts were incubated with 1 μg/ml 4, 6-diamidino-2-phenylindole (DAPI), the GFP and DAPI localization was visualized with a Nikon fluorescent microscope (Exclipse E800) equipped with digital camera. The images obtained at different channels were cropped and merged with imageJ program (National Institutes of Health). The primer sequences are listed in Additional file 6: Table S2.

### Construction of transgenic plants for complementation assay

The promoter region of LUH (2.6 kb), SLK1 (2.4 kb) and SLK2 (1.6 kb) upstream from start codon were PCR amplified from wild type genomic DNA using promoter specific primers with *Sal1* site in the reverse primer. The amplified promoter region of respective genes was cloned in PCR8/GW/TOPO vector (Invitrogen). The coding sequence without stop codon were PCR amplified with gene specific primers using *LUH* (G12254), *SLK1* (G66746) and *SLK2* (G10219) cDNA clones and inserted at *Sal1* site in the promoter containing TOPO vector by In-Fusion HD Cloning Plus. *LUH::LUH*, *SLK1::SLK1* and *SLK2::SLK2* cassettes were transferred into the binary vector pEarleyGate 302, pEarleyGate 301 and pEarleyGate 303 [51] respectively using LR Clonase ll mix (Invitrogen). The binary vector was introduced into *Agrobacterium* strain GV3101 and transformed into mutant plants using floral dip method [52]. The primary transformants were isolated on MS medium with BASTA selection. The resistant plants were confirmed by PCR and RT-PCR to detect T-DNA insertion and gene expression. The primer sequences are listed in Additional file 6: Table S2.

### RNA isolation and quantitative RT-PCR

Total RNA was extracted from 21 day old seedlings using TRIzol reagent (Invitrogen) and purified by RNeasy Plus Mini Kit (Qiagen). For qRT-PCR, 5 μg of DNase treated total RNA was used for cDNA synthesis using oligo (dT) primer and SuperScript III reverse transcriptase (Invitrogen). The target genes were quantified using SYBR Green Supermix reagent (Bio-Rad) with 1:10 dilution of the cDNA and gene specific primers in the Bio-Rad iCycler iQ real time system. *ACTIN2* was used as an internal control for normalization in each quantitative PCR experiment. Real time qRT-PCR was repeated with three biological replicates for each sample. The primer sequences are listed in Additional file 6: Table S2.

### ChIP assay

One gram of 21 day old seedlings was used for ChIP assay. Chromatin preparation and immunoprecipitation were performed as described in [53]. Briefly, the chromatin extracts were prepared from seedlings treated with 1% formaldehyde. The chromatin was sheared to an average length of 500 bp by sonication (ultrasonic processor) and immunoprecipitated with specific antibodies using magnetic protein G beads (Dynabeads protein G, Invitrogen). The antibodies used for ChIP were anti-histone H3 C-terminus (Abcam; 1791), anti-H3K9ac (Abcam; 4441) and anti-H3K14ac (Millipore; 07-353). The immune complexes were washed, eluted from the magnetic protein G beads and reverse cross-linked at 65°C overnight. The DNA was purified using QIAquick PCR purification kit (Qiagen) in a final volume of 50 μL. Three microliter of the DNA was used for each qPCR assay with SYBR Green Supermix reagent (Bio-Rad) in the Bio-Rad iCycler iQ real time system. *ACTIN7* was used as an internal control for normalization in each qPCR experiment. The experiment was repeated with three biological replicates for each sample. The amplification region for the target genes are provided in Additional file 5: Figure S4. The primer sequences are listed in Additional file 6: Table S2.

### Statistical analysis

All experiments were performed at least three times. Error bars in each graph indicate mean values ± SE of three repetitions. P values were determined by Student’s t test.

## Competing interests

The authors declare that they have no competing interests.

## Authors’ contributions

BS, BG and VVS performed the experiments and analyzed the data. VVS conceived the study and wrote the manuscript. All the authors approved the final manuscript.

## Supplementary Material

Additional file 1: Table S1 Effect of salt and osmotic stress on mutant plants. Click here for file

Additional file 2: Figure S1 Complementation assay in *luh-4*, *slk1-1* and *slk2-1* mutant plants. Click here for file

Additional file 3: Figure S2 Quantitative RT-PCR analysis of *SLK1*, *SLK2* and *LUH* genes. Click here for file

Additional file 4: Figure S3 Complementation assay of stress responsive gene expression. Click here for file

Additional file 5: Figure S4 Schematic diagram of target genes *NAC019*, *MYB2* and *RD20*. Click here for file

Additional file 6: Table S2 Oligonucleotide primers used in this study. Click here for file
