# Supplementary material for: Involvement of co-repressor LUH and the adapter proteins SLK1 and SLK2 in the regulation of abiotic stress response genes in Arabidopsis
Source: BMC Plant Biol. 2014 Feb 24;14:54. doi: 10.1186/1471-2229-14-54 (PMC4015341; doi:10.1186/1471-2229-14-54)
Supplement: Additional file 1: Table S1 — Effect of salt and osmotic stress on mutant plants. [file 1471-2229-14-54-S1.pdf]

Table S1 Effect of salt and osmotic stress on mutant plants.

Genotype	Salt stress<br>% Root length<br>Mean ± SE	Salt stress<br>% Fresh weight<br>Mean ± SE	Osmotic stress<br>% Root length<br>Mean ± SE	Osmotic stress<br>% Fresh weight<br>Mean ± SE
seu-1	18 ± 2.2	33 ± 3.2	40 ± 3.6	46 ± 3.7
slk1-1	44 ± 3.6	51± 4.6	66 ± 6.7	76 ± 6.9
slk2-1	32 ± 2.9	46 ± 4.2	56 ± 4.8	63 ± 5.6
luh-4	36 ± 3.3	57 ± 4.8	62 ± 5.8	74 ± 5.8
slk1-1 luh-4	39 ± 4.6	59 ± 6.8	64 ± 5.9	75 ± 6.1
slk2-1 luh-4	38 ± 4.6	59 ± 4.9	61 ± 5.5	69 ± 5.8

The plants were grown in medium for six days and transferred to medium supplemented with 125 mM NaCl and 300 mM mannitol for salt and osmotic stress treatment respectively. The root length and fresh weight is expressed as a percentage relative to plants grown on MS medium without stress treatment and plants grown on the medium with 125 mM NaCl and 300 mM mannitol after 15 and 25 days respectively**.** SE was determined from 20 – 25 plants per replicate (n = 4).
