# Supplementary material for: Involvement of co-repressor LUH and the adapter proteins SLK1 and SLK2 in the regulation of abiotic stress response genes in Arabidopsis
Source: BMC Plant Biol. 2014 Feb 24;14:54. doi: 10.1186/1471-2229-14-54 (PMC4015341; doi:10.1186/1471-2229-14-54)
Supplement: Additional file 2: Figure S1 — Complementation assay in luh-4, slk1-1 and slk2-1 mutant plants. [file 1471-2229-14-54-S2.pdf]

Figure S1

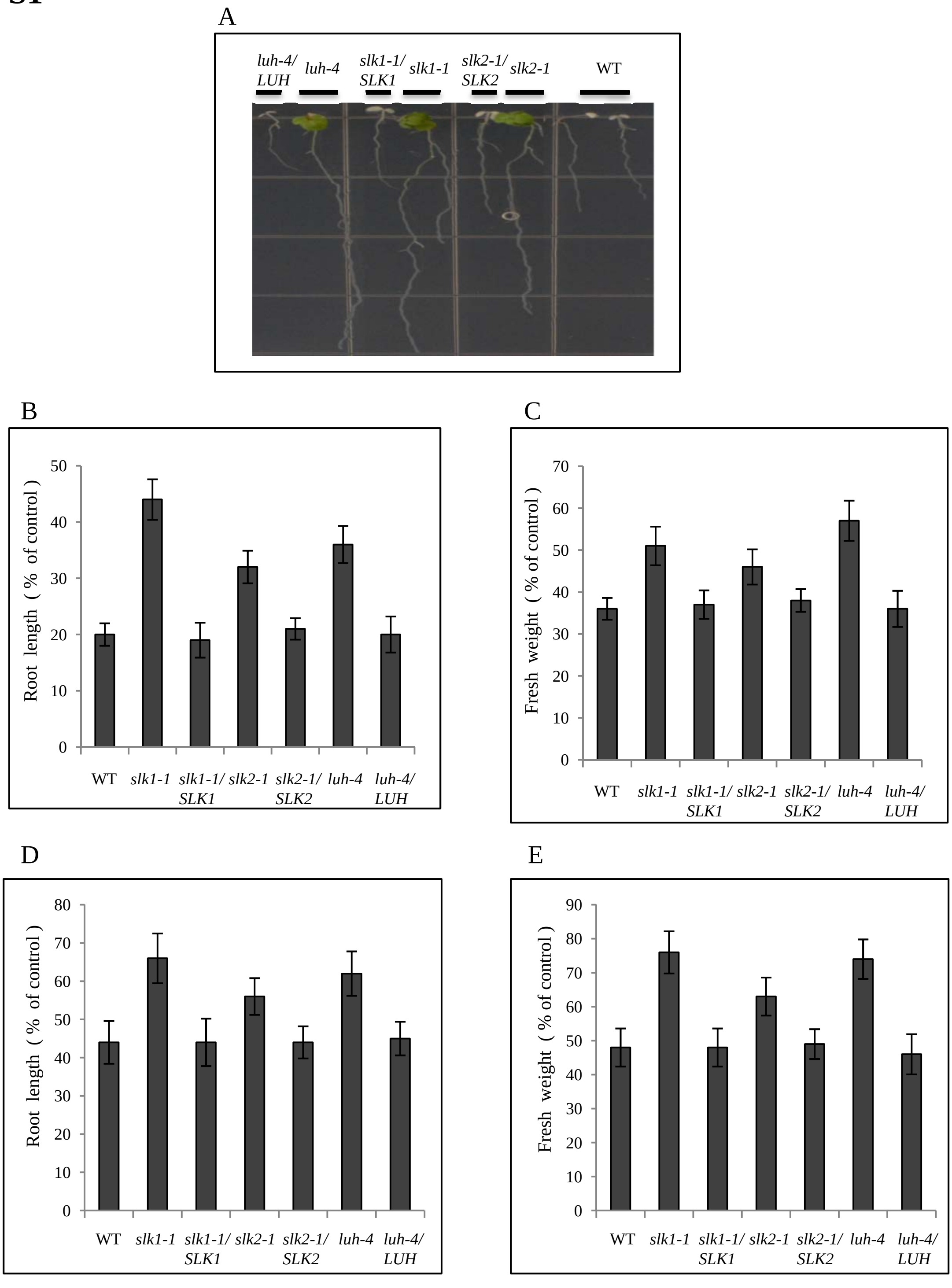

FIGURE S1: Complementation assay in *luh-4, slk1-1* and *slk2-1* mutant plants.

(A) *SLK1*, *SLK2* and *LUH* (with its native promoter and coding sequence) were transformed into mutant plants. The single mutants and a representative homozygous line with wild type gene were grown on MS medium for six days. The plants were transferred to MS medium supplemented with 125 mM NaCl for salt stress treatment and grown in growth chamber for 15 days. **(B)** Root length of plants grown on MS medium with 125 mM NaCl for 15 days. **(C)** Fresh weight of plants grown on MS medium with 125 mM NaCl for 15 days. **(D)** Root length of plants grown on MS medium with 300 mM mannitol for 25 days. **(E)** Fresh weight of plants grown on MS medium with 300 mM mannitol for 25 days. The root length and fresh weight for salt and osmotic stress is presented as a percentage relative to plants grown on MS medium without stress treatment. Error bars are SE with 10 – 15 plants per replicate (n = 4). Complemented plants are denoted as *slk1-1*/*SLK1*, *slk2-1*/*SLK2* and *luh-4*/*LUH.*
