# Supplementary material for: Involvement of co-repressor LUH and the adapter proteins SLK1 and SLK2 in the regulation of abiotic stress response genes in Arabidopsis
Source: BMC Plant Biol. 2014 Feb 24;14:54. doi: 10.1186/1471-2229-14-54 (PMC4015341; doi:10.1186/1471-2229-14-54)
Supplement: Additional file 3: Figure S2 — Quantitative RT-PCR analysis of SLK1, SLK2 and LUH genes. [file 1471-2229-14-54-S3.pdf]

FIGURE S2

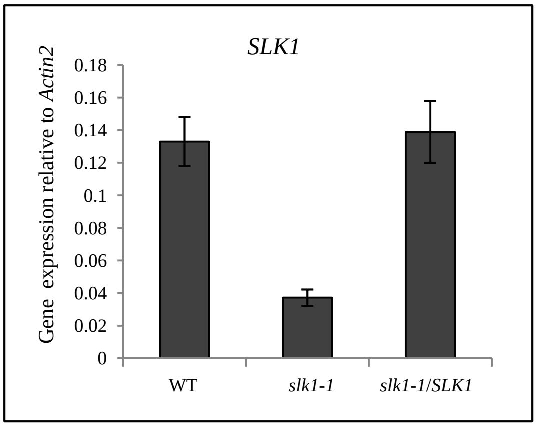

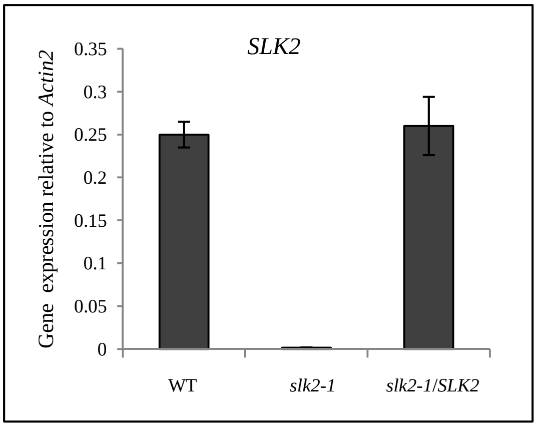

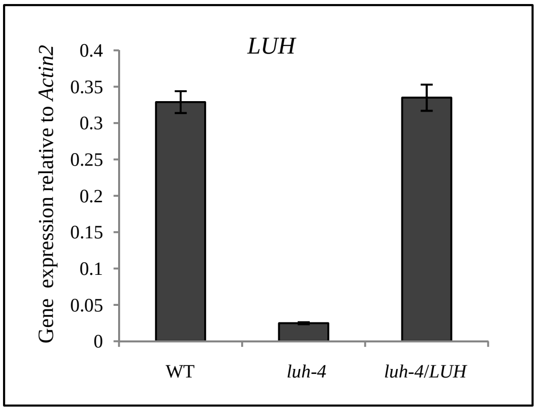

FIGURE S2: Quantitative RT-PCR analysis of *SLK1*, *SLK2* and *LUH* genes.
Transcript levels of *SLK1*, *SLK2* and *LUH* were quantitated using qRT-PCR. *ACTIN2* served as an internal control in the wild type, mutant and complemented plants. The indicated gene expression is relative to *Actin2* from three biological replicates. Error bars are SE (n = 3). Complemented plants are denoted as *slk1-1*/*SLK1*, *slk2-1*/*SLK2* and *luh-4*/*LUH*
