# Supplementary material for: Involvement of co-repressor LUH and the adapter proteins SLK1 and SLK2 in the regulation of abiotic stress response genes in Arabidopsis
Source: BMC Plant Biol. 2014 Feb 24;14:54. doi: 10.1186/1471-2229-14-54 (PMC4015341; doi:10.1186/1471-2229-14-54)
Supplement: Additional file 4: Figure S3 — Complementation assay of stress responsive gene expression. [file 1471-2229-14-54-S4.pdf]

FIGURE S3

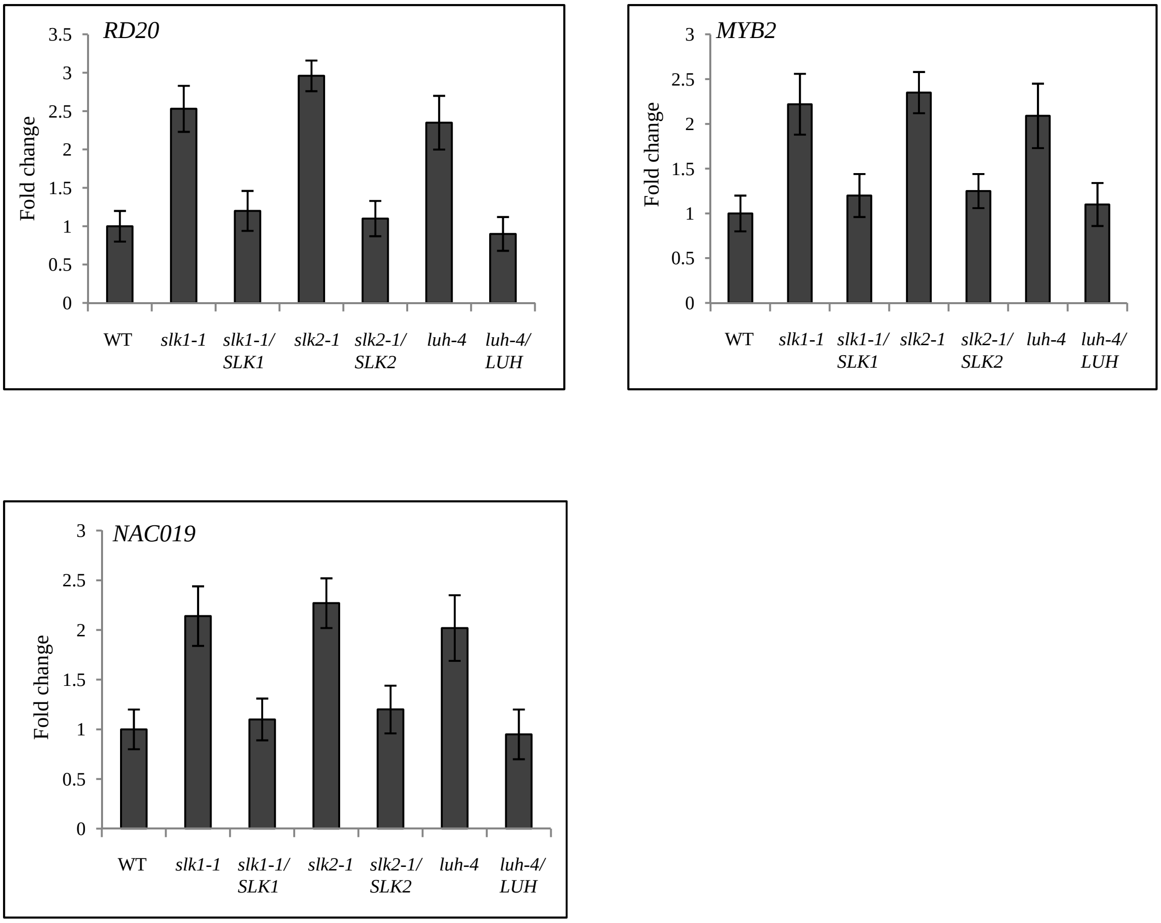

FIGURE S3: Complementation assay of stress responsive gene expression.
Transcript levels of *RD20*, *MYB2* and *NAC019* were quantitated using qRT-PCR. A*CTIN2* served as an internal control in the wild type, complemented plants. SE (*n* = 3). Complemented plants are denoted as *slk1-1*/*SLK1*, *slk2-1*/*SLK2* and *luh-4*/*LUH*
