# Supplementary material for: Involvement of co-repressor LUH and the adapter proteins SLK1 and SLK2 in the regulation of abiotic stress response genes in Arabidopsis
Source: BMC Plant Biol. 2014 Feb 24;14:54. doi: 10.1186/1471-2229-14-54 (PMC4015341; doi:10.1186/1471-2229-14-54)
Supplement: Additional file 5: Figure S4 — Schematic diagram of target genes NAC019, MYB2 and RD20. [file 1471-2229-14-54-S5.pdf]

**Figure S4** **Schematic diagram of target genes *NAC019, MYB2* and *RD20*.**

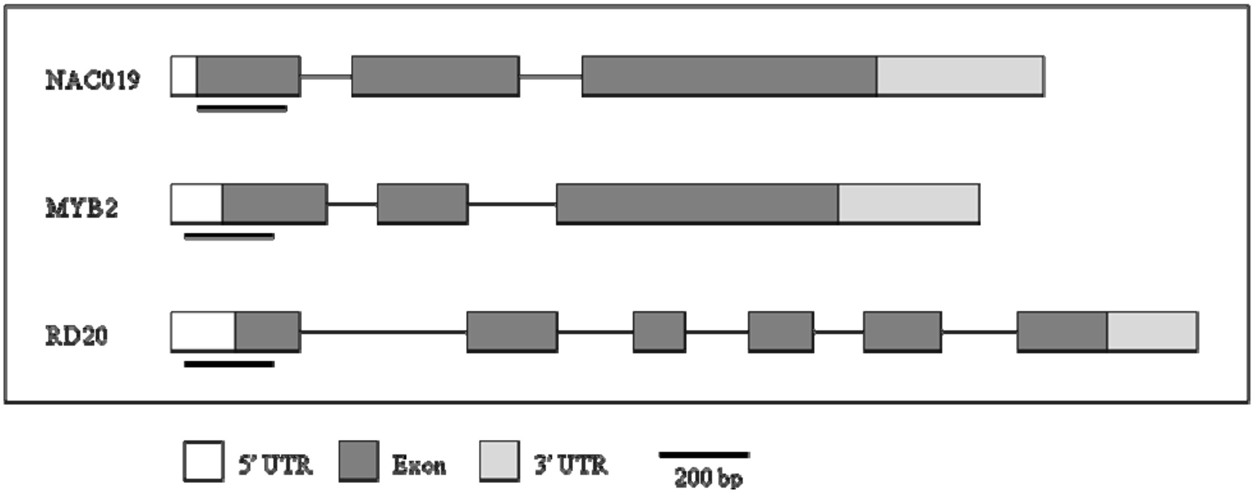


The thin line denotes intron and the dark line underneath the gene denotes the amplification region for ChIP analysis.
