# Supplementary material for: Involvement of co-repressor LUH and the adapter proteins SLK1 and SLK2 in the regulation of abiotic stress response genes in Arabidopsis
Source: BMC Plant Biol. 2014 Feb 24;14:54. doi: 10.1186/1471-2229-14-54 (PMC4015341; doi:10.1186/1471-2229-14-54)
Supplement: Additional file 6: Table S2 — Oligonucleotide primers used in this study. [file 1471-2229-14-54-S6.pdf]

Table S2 Oligonucleotide primers used in this study.

Yeast Two Hybrid Assay.

LUH_BD_INFU_F	AGGAGGACCTGCATATGATGGCTCAGAGTAATTGGGAA
LUH_BD_INFU_R	GCCGCTGCAGGTCGACCTACTTCCAAATCTTTACGGAT
LUFS_BD_INFU_R	GCCGCTGCAGGTCGACCTACCTTGCAATGAAAATGTCCCA
SLK1_BD_INFU_F	AGGAGGACCTGCATATGATGAACAGAACGGTGGTCTCG
SLK1_BD_INFU_R	GCCGCTGCAGGTCGACTTACAAGCCACCATAGATATC
SLK1_AD_INFU_F	CAGATTACGCTCATATGATGAACAGAACGGTGGTCTCG
SLK1_AD_INFU_R	CGAGCTCGATGGATCCTTACAAGCCACCATAGATATC
SLK2_BD_INFU_F	AGGAGGACCTGCATATGATGGCTTCTTCAACTTCTGGG
SLK2_BD_INFU_R	GCCGCTGCAGGTCGACTCATGACTTCCAAGAATATCC
SLK2_AD_INFU_F	CAGATTACGCTCATATGATGGCTTCTTCAACTTCTGGG
SLK2_AD_INFU_R	CGAGCTCGATGGATCCTCATGACTTCCAAGAATATCC
H3_AD_INFU_F	CAGATTACGCTCATATGATGGCTCGTACCAAGCAAACC
H3_AD_INFU_R	CGAGCTCGATGGATCCTTAAGCCCTCTCGCCTCTAAT
H2B_AD_INFU_F	CAGATTACGCTCATATGATGGCACCAAGAGCCGAGAAG
H2B_AD_INFU_R	CGAGCTCGATGGATCCTCAAGAGCTAGTAAACTTGGT

Repression Assay.

CaMV_pUASluc2F	CTCGGCGGCCAAGCTTGAGACTTTTCAACAAAGGG
CaMV_pUASluc2R	ATCTGTTAACGAATTTCCTCTCCAAATGAAATGAA
5xGal4DBF	GAGGATATCAAGATCCCAAGCTTGCATGCCTGCAGGT
5xGal4DBR	CGCCGAGGCCAGATCCGGCGCTCGCTAGAGTCTCCG
CaMV_PRLnullF	TCGACGCGTAGAATTTGCCTGCAGGTCAACATGGTG
CaMV_PRLnullR	CTCACGACCAACTTCCTAGAGTCGAGGTCCTCTCC
pGBTK_GAL4F	TACTCGAGGGGGATCAAGCTTGAAGCAAGCCTCCT

pGBTK_GAL4R	ATTCGCTAGTGGATCAAGCTTGCATGCCGGTAGAG
LUH_pXSNF	TACTCGAGGGGGATCATGGCTCAGAGTAATTGGGAA
LUH_pXSNR	ATTCGCTAGTGGATCCTACTTCCAAATCTTTACGGA
LUFS_pXSNR	ATTCGCTAGTGGATCCTACTCCTTCGCTTTACCTTGTT

Nuclear Localization: GFP Fusion.

LUH_gfpF	CGCGGGCCCGGGATCCATGGCTCAGAGTAATTGGGA
LUH_gfpR	TAGATCCGGTGGATCCTACTTCCAAATCTTTACGGA
SLK1_gfpF	CGCGGGCCCGGGATCCATGAACAGAACGGTGGTCTC
SLK1_gfpR	TAGATCCGGTGGATCTTACAAGCCACCATAGATATC
SLK2_gfpF	CGCGGGCCCGGGATCCATGGCTTCTTCAACTTCTGG
SLK2_gfpR	TAGATCCGGTGGATCTCATGACTTCCAAGAATATC

Split Luciferase Complementation Assay.

LUH_NlucF	GGTACCATGGCTCAGAGTAATTGGGAA
LUH_NlucR	GTCGACCTTCCAAATCTTTACGGATTTG
LUFS_NlucR	GTCGACCTCCTTCGCTTTACCTTGTT
SLK1_ClucF	GGTACCATGAACAGAACGGTGGTCTCG
SLK1_ClucR	CTGCAGTTACAAGCCACCATAGATATC
SLK2_ClucF	GGTACCATGGCTTCTTCAACTTCTGGG
SLK2_ClucR	CTGCAGTCATGACTTCCAAGAATATCC
H3_ClucF	GGTACCATGGCTCGTACCAAGCAAACC
H3_ClucR	CTGCAGTAAGCCCTCTCGCCTCTAAT
H2B_ClucF	GGTACCATGGCACCAAGAGCCGAGAAG
H2B_ClucR	CTGCAGTCAAGAGCTAGTAAACTTGGT

qRT-PCR.

RD20RT-PCRF	CCGAAGGAAGGTATGTCCCA
RD20RT-PCRR	GTTTGCGAGAATTGGCCCTC
MYB2RT-PCRF	CAACGATTGGGGCTGTGTTG
MYB2RT-PCRR	TCAGGGGATTAAAACAAGAGAGGA
NAC019RT-PCRF	TAACCCAAACCGCATCTCGT
NAC019RT-PCRR	ACTTGCCCCGAATACCCAAA
ACT2RT-PCRF	GATCTCCAAGGCCGAGTATGAT
ACT2RT-PCRR	CCCATTCATAAAACCCCAGC
SLK1RT-PCRF	TGGCTCAACGGGCAATAACACCA
SLK1RT-PCRR	CTGACTCGAGAACCCCTCTTGTTTAC
SLK2RT-PCRF	CCGGAGTGCATCCCCGAGCTATC
SLK2RT-PCRR	TCTGTTTCTTCCCATGTTGGTATTGC
LUHRT-PCRF	CGTTTCCAGCCAAGAACAGGACAG
LUHRT-PCRR	CCACAGCTCTATAGCCTGATAGCC

ChIP Assay.

RD20ChIPF	GCATAAGAAGAAACAGAGAGCATTAT
RD20ChIPR	ATTCACACTTACATGGTTTTGGTAAT
MYB2ChIPF	CCACAAAACCATTCACACCATC
MYB2ChIPR	CATGGATAGAGACGAAGTTGAC
NAC019ChIPF	AAAAATATGGGTATCCAAGAAACTGA
NAC019ChIPR	GTAAAACCCATGGATCGAATTTGTA
ACT7F	CGTTTCGCTTTCCTTAGTGTTAGCT
ACT7R	AGCGAACGGATCTAGAGACTCACCTTG

Complementation Assay.

SLK1 promoterF	CGGAAAGATGTATGGGCTTCGGCCCA
SLK1 promoterR	GCCCCACCCACGAGAGCAGATAACAG
SLK2 promoterF	GTTATGTCTCATTATCAGTCCATCACAG
SLK2 promoterR	TAAAGGGTAGGTCCCAAGTGAGTCTG
LUH promoterF	AGTGAAGCTTAGTCTTGACCTCTATA
LUH promoterR	AGCTTCAGCCCAAGATCGAGCTGC
SLK1CDSF	TGGGTGGGGCGTCGACATGAACAGAACGGTGGTCTCGG
SLK1CDSR	ATTCGCCCTTGTCGACCAAGCCACCATAGATATCATTG
SLK2CDSF	CTACCCTTTAGTCGACATGGCTTCTTCAACTTCTGGGA
SLK2CDSR	ATTCGCCCTTGTCGACTGACTTCCAAGAATATCCTCC
LUHCDSF	GGCTGAAGCTGTCGACATGGCTCAGAGTAATTGGGAAG
LUHCDSR	ATTCGCCCTTGTCGACCTTCCAAATCTTTACGGATTTG
